# Supplementary material for: Taxonomic revision of the tarantula genus Aphonopelma Pocock, 1901 (Araneae, Mygalomorphae, Theraphosidae) within the United States
Source: Zookeys. 2016 Feb 4;(560):1–340. doi: 10.3897/zookeys.560.6264 (PMC4768370; doi:10.3897/zookeys.560.6264)
Supplement: Supplementary material 2 — Compressed files (.zip) containing Aphonopelma boxplot and PCA morphospace data. [file zookeys-560-001-s002.zip › Supp2a.zip!/Appendix 2A/PCA_morphospace/Aphonopelma_PCA_males/Pygmy/males_pygmy_PC2~PC3.pdf]

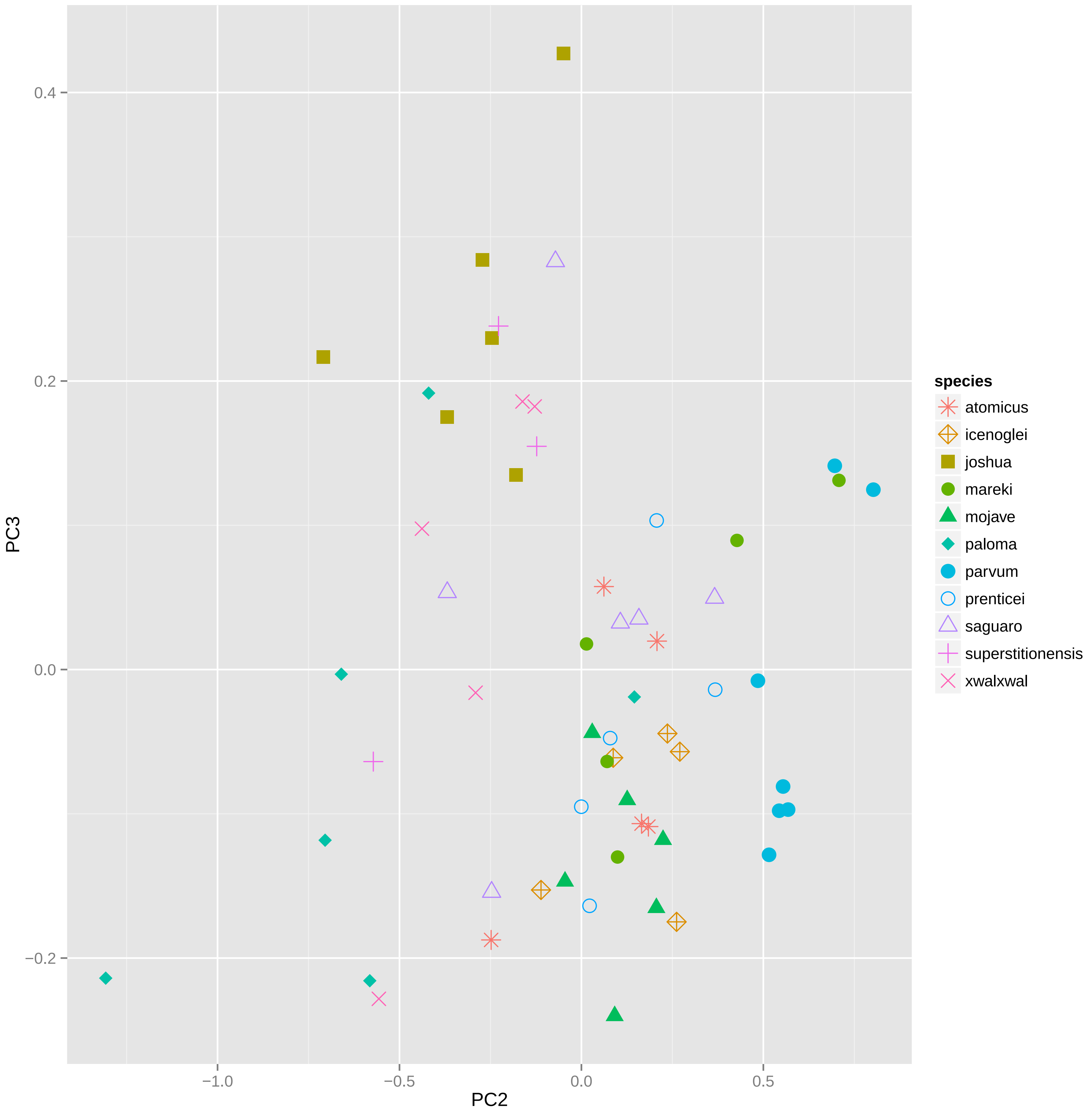

PC3
PC2
0.4
0.2
0.0
−0.2
−1.0
−0.5
0.0
0.5
species
atomicus
icenoglei
joshua
mareki
mojave
paloma
parvum
prenticei
saguaro
superstitionensis
xwalxwal
